# Results of a randomized, placebo‐controlled phase 1b study in MCI and mild AD patients with the orally available PRI‐002 with a unique mode of action.

**DOI:** 10.1002/alz70859_101508

**Published:** 2025-12-25

**Authors:** Dieter Willbold, Janine Kutzsche, Nicoleta Carmen Cosma, Gunther Kauselmann, Dagmar Jürgens, Oliver Peters

**Affiliations:** ^1^ Priavoid, Jülich Germany; ^2^ Forschungszentrum Julich, Julich, Priavoid GmbH Germany; ^3^ Heinrich‐Heine‐Universität Düsseldorf, Düsseldorf, NRW Germany; ^4^ Forschungszentrum Julich, Julich, NRW Germany; ^5^ Department of Psychiatry and Neuroscience, Memory Clinic, Charité‐Universitätsmedizin Berlin, Campus Benjamin Franklin, Berlin, Berlin Germany; ^6^ Forschungszentrum Jülich, Jülich Germany; ^7^ Priavoid, Düsseldorf, NRW Germany; ^8^ Charité – Universitätsmedizin Berlin, corporate member of Freie Universität Berlin and Humboldt‐Universität zu Berlin, Berlin Germany

## Abstract

**Background:**

Self‐replicating amyloid beta (Aβ) oligomers are described to be synaptotoxic and responsible for reduced synaptic plasticity, impaired neuronal function and thus for development and progression of Alzheimer’s disease (AD). The all‐D‐enantiomeric peptide PRI‐002 was developed to disassemble toxic Aβ oligomers into harmless Aβ monomers, very similar to a chaperone. PRI‐002 is expected to reduce neurotoxicity and to restore synaptic plasticity in early AD stages. Target engagement has been demonstrated in vitro, in vivo and ex vivo. PRI‐002 has previously been shown to reverse cognition deficits in four different animal models in four different laboratories. PRI‐002 has also demonstrated to be safe and well tolerable in healthy volunteers. Here we aim to demonstrate safety in patients with mild cognitive impairment (MCI) and mild dementia due to AD and to explore efficacy.

**Method:**

We carried out a randomized, placebo‐controlled, double‐blind, Phase 1b study to evaluate safety, tolerability and pharmacodynamics of PRI‐002 in 20 patients with MCI to mild dementia due to AD. Eligible patients were blindly randomly assigned (1:1) to receive 300 mg PRI‐002 per day or placebo for 28 days. Follow‐up assessment took place on day 56. The trial is registered in EudraCT 2020‐003416‐27.

**Result:**

19 out of 20 patients were randomly assigned to PRI‐002 (n=9) or placebo (n=10) and completed the study per protocol. PRI‐002 was well tolerated. No SAEs were reported. Clinically meaningful findings were not reported. ECG, EEG and MRI revealed no changes. As expected, no ARIA were observed. No significant changes were detected in p‐tau, t‐tau, Aβ 1‐40, Aβ 1‐42 and Aβ oligomers in CSF. In contrast to patients in the placebo group, each of the patients in the verum group had increased short‐term‐memory abilities as demonstrated in the CERAD word list at day 56 vs. baseline (p<0.01).

**Conclusion:**

PRI‐002 was well tolerated. No biomarker changes have been found after 28 days of treatment. No ARIA were detected. Memory improved significantly in the verum group. The randomized, double‐blind, placebo‐controlled PRImus‐AD phase 2 study has finished recruiting to assess safety and efficacy of PRI‐002 in patients with MCI and mild dementia due to AD (EU CT# 2022‐503148‐41).

